# A Comparison of Neuroimaging Findings in Childhood Onset Schizophrenia and Autism Spectrum Disorder: A Review of the Literature

**DOI:** 10.3389/fpsyt.2013.00175

**Published:** 2013-12-20

**Authors:** Danielle A. Baribeau, Evdokia Anagnostou

**Affiliations:** ^1^Department of Psychiatry, University of Toronto, Toronto, ON, Canada; ^2^Autism Research Centre, Bloorview Research Institute, University of Toronto, Toronto, ON, Canada

**Keywords:** autism spectrum disorder, childhood onset schizophrenia, neuroimaging, magnetic resonance imaging, child development, review

## Abstract

**Background**: Autism spectrum disorder (ASD) and childhood onset schizophrenia (COS) are pediatric neurodevelopmental disorders associated with significant morbidity. Both conditions are thought to share an underlying genetic architecture. A comparison of neuroimaging findings across ASD and COS with a focus on altered neurodevelopmental trajectories can shed light on potential clinical biomarkers and may highlight an underlying etiopathogenesis.

**Methods**: A comprehensive review of the medical literature was conducted to summarize neuroimaging data with respect to both conditions in terms of structural imaging (including volumetric analysis, cortical thickness and morphology, and region of interest studies), white matter analysis (include volumetric analysis and diffusion tensor imaging) and functional connectivity.

**Results**: In ASD, a pattern of early brain overgrowth in the first few years of life is followed by dysmaturation in adolescence. Functional analyses have suggested impaired long-range connectivity as well as increased local and/or subcortical connectivity in this condition. In COS, deficits in cerebral volume, cortical thickness, and white matter maturation seem most pronounced in childhood and adolescence, and may level off in adulthood. Deficits in local connectivity, with increased long-range connectivity have been proposed, in keeping with exaggerated cortical thinning.

**Conclusion**: The neuroimaging literature supports a neurodevelopmental origin of both ASD and COS and provides evidence for dynamic changes in both conditions that vary across space and time in the developing brain. Looking forward, imaging studies which capture the early post natal period, which are longitudinal and prospective, and which maximize the signal to noise ratio across heterogeneous conditions will be required to translate research findings into a clinical environment.

## Introduction

Autism spectrum disorder (ASD) is a neurodevelopmental disorder of increasing prevalence in the modern era. Presently, this condition is reported to affect 1 in 88 individuals (1). Manifested by social communication deficits and restricted or repetitive interests and behaviors, children with ASD present along a wide spectrum of clinical severity, from mild social difficulties to severe functional impairment. This condition typically presents in the first 3 years of life, manifested by a failure to gain, or a loss of, social communication milestones.

Childhood onset schizophrenia (COS), on the other hand, is a relatively rare disorder, affecting 1 in 10,000–30,000 children (2). The diagnostic criteria are the same as in adult onset schizophrenia, including the presence of positive and/or negative symptoms (3), but with onset occurring prior to the 13th birthday (4). Despite clinical heterogeneity, COS typically presents with psychotic symptoms after age seven, and is associated with a more severe course and poorer outcomes as compared to adult onset schizophrenia (2).

Although presently considered to separate clinical entities, prior to the twentieth century, catatonia, social withdrawal, bizarre behavior, and/or psychosis in children were considered undifferentiated conditions, labeled as “hereditary insanity,” “dementia praecox,” or “developmental idiocy” (5). With the onset of contemporary nosology, “autistic behavior and social withdrawal” were initially specified as features of “childhood schizophrenia” in the first and second editions of the Diagnostic and Statistical Manual of Mental Disorder (DSM-I and -II). Although formally defined as separate entities in DSM-III (6), at present the DSM-5 permits concurrent diagnosis of both conditions, should an individual with ASD subsequently develop prominent delusions or hallucinations (3).

In the current review, a comparison between ASD and COS was chosen for several reasons. Firstly, children with co-occurring and overlapping symptoms complicate a diagnosis (2, 4). At times, a period of medication washout and inpatient observation is required to achieve a diagnostic consensus (7), further supporting a need for brain based biomarkers of disease state and treatment response. Indeed, over one quarter of patients diagnosed with COS display prodromal neurodevelopmental disturbances, meeting criteria for pervasive developmental disorder, or ASD (8, 9). Children diagnosed with ASD are more likely to report psychotic symptoms in adolescence and adulthood (10, 11), although the exact incidence of a subsequent diagnosis of schizophrenia varies by study, ranging from 0 to 7% (12–14). From a neuroimaging perspective, analysis of atypical brain “growth curves” may afford an opportunity for early identification and risk stratification; consistent with the present goal of moving toward biologically based diagnostic categories in neuropsychiatric disease.

Secondly, a growing body of literature supports a neurodevelopmental origin of both schizophrenia and autism, with a shared genetic architecture contributing to, or precipitating, the development of both conditions (15, 16). Some have hypothesized that ASD and schizophrenia are diametrically opposed with respect to underlying pathology (17). While adult onset schizophrenia and ASD have been compared in previous reviews [see Ref. (18)], a focus on COS specifically permits a more in-depth analysis of aberrant neurodevelopmental trajectories across comparable age ranges, which may provide insight into disease pathogenesis.

This review intends to translate several decades of neuroimaging research for a clinical audience, to highlight our current understanding of similarities and differences in the clinicopathogenesis of ASD and COS from a neuroimaging perspective. To our knowledge, this is the first focused review of neuroimaging findings in ASD and COS.

## Structural MRI Studies (Volumetric Analysis, Cortical Thickness and Morphology, and Region of Interest Studies)

### Volumetric analysis

Structural magnetic resonance imaging (MRI) analysis for neuropsychiatric diseases began to emerge in the 1990s. Early trials employed manual delineation of gray and white matter to investigate specific regions of interest. With advancement in high resolution MRI technology and automated analysis, voxel-based morphometry (VBM) made it possible to quantify the specific gray matter content of each voxel (a volumetric pixel) in an image, allowing large data sets to be processed more efficiently (19). For statistical comparisons between case and control populations, images are “warped” onto a common template, and the degree of transposition of each voxel can be quantified. Inferences must be heeded with the consideration that the relative volumetric differences by region can vary by age, gender, whole brain volume, and by IQ, thus the degree to which these factors have been controlled for must be kept in mind.

#### Volumetric analysis in COS

Initial trials conducted by the National Institute of Mental Health (NIMH) on a cohort of children with COS, identified a pattern of reduced cerebral volumes and larger ventricles, consistent with findings in the adult onset schizophrenia population (20). With expansion and longitudinal analysis of this patient sample, investigators were able to localize and describe patterns of change in brain structure and volume over time. While typically developing children were found to have a small decrease in cortical gray matter (~2%) in the frontal and parietal regions throughout adolescence, children with COS displayed exaggerated gray matter losses (~8%), involving the frontal, parietal, and temporal lobes. Of note, baseline IQ varied significantly between case and control groups in this data set (70 vs. 124) (21).

Subsequent analysis on the same NIMH sample (*n* = 60 patients), suggested that this pattern took on a “back to front” trajectory, with losses originating in the parietal lobes and spreading anteriorly over time (22). This pattern persisted after controlling for IQ and medication administration (23). Despite significant differences at an early age, the rate of gray matter loss was shown to level off in early adulthood, implicating adolescent neurodevelopment as a key window in disease pathogenesis (22, 24). This data is consistent with hypotheses pertaining to exaggerated synaptic pruning as a feature of schizophrenia (25).

Later work by the same group demonstrated that the above-described pattern was specific for COS. Using VBM, 23 COS patients were compared to 38 age and gender matched healthy control subjects and 19 patients with other psychotic symptoms but not meeting criteria for COS, defined as “multidimensionally impaired” (MDI). MRI scans were conducted at study intake, and at 2.5 years follow up. The MDI group had equal exposure to neuroleptics at study intake, and had a similar degree of cognitive impairment. Total gray matter loss between the two time points demonstrated 5.1% loss for COS patients, 0.5% loss for MDI patients, and 1.5% loss for healthy control subjects. Thus, exaggerated gray matter loss during adolescence was considered to be a potential biomarker of COS (26).

There is very little literature looking at infants or toddlers who subsequently develop schizophrenia, given the methodological complexities of such a study. That being said, offspring of mothers with schizophrenia were found on average to have *larger* intracranial volumes, greater volumes of CSF, and greater gray matter volume on structural MRI in male neonates, compared to controls, although controlling for total intracranial volume resulted in all differences being non-significant (27).

#### Volumetric analysis in ASD

**Figure 1:**
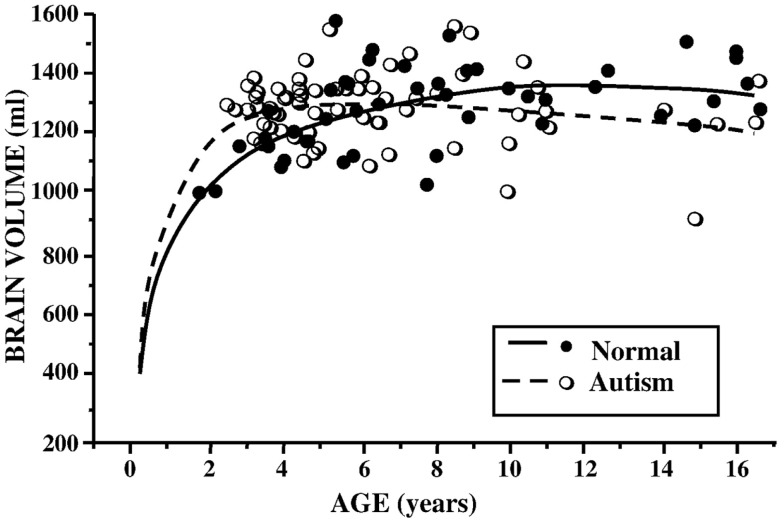
**Brain volume (milliliters) by age (years) in children with ASD and controls**. Reproduced with permission from Courchesne et al. (37), adapted from Courchesne et al. (32).

In ASD, earlier studies suggested a pattern of increased total brain volume, as well as increased ventricle size (28–30). Analyses across age ranges helped to further elucidate the chronology of this brain overgrowth picture. Indeed, exaggerated gray and white matter volumes seemed most pronounced in younger children, while older children with ASD had more typically appearing brains, when compared to their peers (31, 32) (see Figure 1). The hypothesis of brain overgrowth correlated with the measureable increase in rate of growth of head circumference during the first few years of life as well in this population (33, 34).

In 2005, a meta-analysis of published data on brain volume, head circumference, and post-mortem brain weight in ASD, further described the effect of age, with most marked differences occurring in the first few years of life. In adulthood, however, brain sizes did not vary from controls (35). Subsequent longitudinal and cross-sectional data from hundreds of children and adults with ASD documented volume enlargement during preschool years, most prominently in the anterior regions, followed by possible growth arrest or exaggerated losses later in childhood (36–38). Using cross-sectional age-adjusted data, Schumann et al. (36), for example, showed that children with ASD had 10% greater white matter volume, 6% greater frontal gray matter volume, and 9% greater temporal gray matter volume at 2 years of age. Longitudinal data showed altered growth trajectories at follow up scans (36).

Volumetric differences did not hold true in all ASD studies however, for example, when structural MRI from children with ASD were compared to children with other developmental delays (39, 40). Similarly, a recent systematic review of published data on head circumference overgrowth in children with ASD suggests differences may be much more subtle than previously thought. The authors attribute exaggerated differences to biased normative data in the CDC head circumference growth curves, to the selection of control groups from non-local communities, as well as to a failure to control for head circumference confounders such as weight and ethnicity (41).

**Figure 2:**
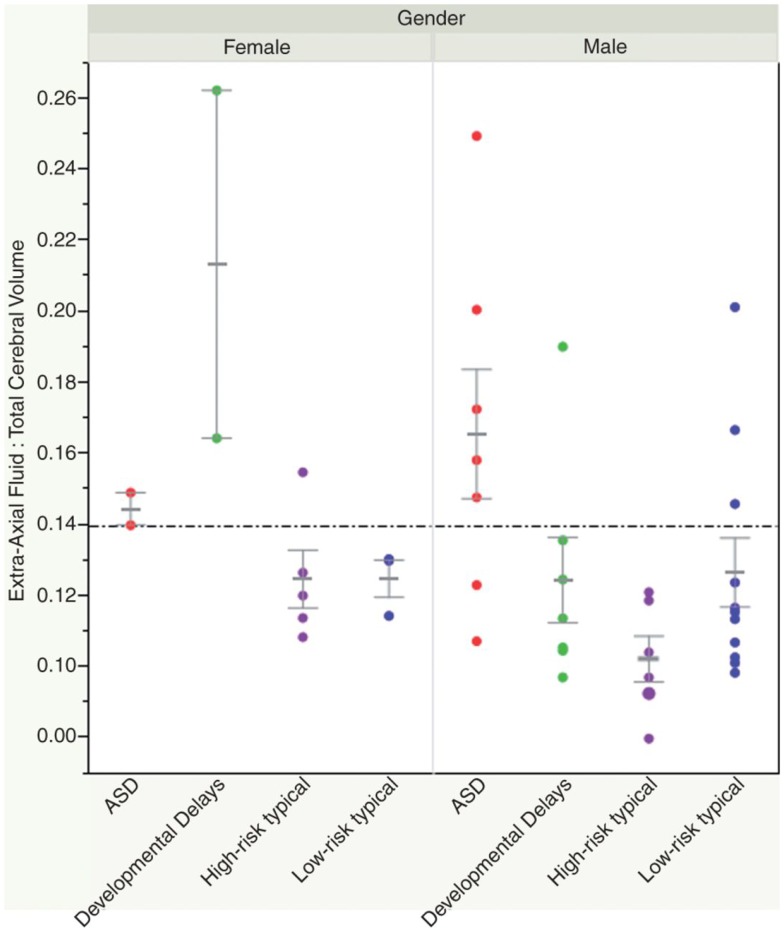
**Shen et al. (42) showed how an elevated ratio of fluid:brain volume (above 0.14) at 12–15 months of age was predictive of a subsequent diagnosis of ASD, with 78% sensitivity and 79% specificity in their sample**. Reproduced with permission from Shen et al. (42).

Recently, a small study looked at whether volumetric MRI might be predictive of a subsequent diagnosis of ASD, prior to the development of clinical symptoms. A group of 55 infants (33 of which were considered high risk given that they had a sibling with ASD) were scanned prospectively at three time points prior to 24 months of age. At 24 and 36 months, they underwent detailed developmental assessments, at which point 10 infants were identified as having a diagnosis of ASD, and 11 were noted to have other developmental delays. The authors found increased extra-axial fluid volume in infants who developed ASD, and quantified the difference through manual delineation of CSF compartments. They were able to show that a ratio of fluid:brain volume of >0.14 yielded 79% specificity and 78% sensitivity in 12–15 month old infants regarding a subsequent diagnosis of ASD (42) (see Figure 2). The finding remains to be replicated.

##### Summary and comparison

In summary, volumetric analyses in ASD describe early brain overgrowth in the first few years of life, a finding that is difficult to contrast to COS, given the methodological complexity of acquiring neuroimaging data in very young children or neonates who subsequently develop this condition. During childhood and adolescence, volumetric data suggests that individuals with ASD may have attenuated brain growth or exaggerated volume loss, since adults with ASD have comparable brain volumes to their typically developing peers. Some similarities emerge with the COS population, given findings of exaggerated gray matter loss during adolescent years.

### Cortical thickness and morphology

With advancements in computational statistics, it became possible extract a more detailed analysis of the cortical gray matter with respect to surface morphology. Specifically, the transposition of cortical imaging data onto a common surface template allowed cortical gray matter volume to be further quantified in terms of cortical thickness, surface area, and gyrification. More recently, complex statistical approaches employing mathematical algorithms and machine-learning models have manipulated neuroimaging data collected from both volumetric and cortical thickness measurements, in efforts to generate diagnostic classifiers of ASD/COS.

Cortical measurements are of interest for neurodevelopmental disorders as they are thought to represent distinct embryological processes under tight regulatory control (43). Cortical surface area, for example, reflects to the process of neural stem cell proliferation and migration early in embryologic development (44). Cortical thickness, on the other hand, reflects axon and dendrite remodeling, myelination, and synaptic pruning, in a dynamic process lasting from birth into adulthood (45).

#### Cortical thickness and morphology in COS

**Figure 3:**
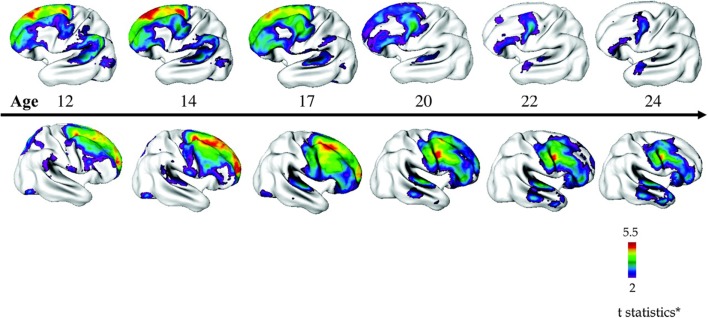
**Progressive loss of cortical thickness in a “front to back” pattern observed through longitudinal imaging of 70 children with COS compared to 72 control participants**. Reproduced with permission from Gogtay (160), adapted from Greenstein et al. (46).

In the NIMH-COS sample (46), a combination of cross-sectional and longitudinal data from 70 patients compared to controls revealed diffuse decreases in mean cortical thickness in childhood (~7.5% smaller), which became localized specifically to the frontal and temporal lobes with increasing age. Statistical significance survived correction for covariates such as sex, socioeconomic status, and IQ. Accordingly, while individuals with COS displayed global gray matter and cortical thickness losses in childhood, with age these losses became similar to those observed in adult onset schizophrenia, with deficits localizing more anteriorly (see Figure 3).

Interestingly, in two separate samples, non-affected siblings of COS probands also demonstrated a pattern of decreased cortical thickness in the frontal, temporal and parietal lobes during childhood and adolescence, which then normalized in early adulthood, implicating some sort of compensatory mechanism despite underlying genetic risk (47, 48).

With hospitalization and medication management, symptom remission correlated with localized increases in cortical thickness measurable in specific subregions of the cortex (49), irrespective of choice of antipsychotic (50). Children who had other psychiatric conditions with comorbid psychotic symptoms but not meeting full criteria for COS demonstrated cortical deficits in prefrontal/temporal pattern as well, but deficits were smaller and less striking than in COS patients (51).

As mentioned in the introduction to this section, complex algorithms and mathematical protocols have been designed to identify and combine measurements that may be predictive of disease state. A multivariate machine-learning algorithm applied to cortical thickness data from the NIMH cohort was able to correctly classify 73.7% of patients with COS and controls. Through this method, 74 “important” regions were identified. Areas with the most predictive power clustered in frontal regions (primarily the superior and middle frontal gyris), and the left temporoparietal region (52). Given the rarity of COS in the general population, and the case-control study design, these results were not validated in a separate study population, precluding any calculation of positive or negative predictive value, and thus limiting any inferences regarding clinical utility.

#### Cortical thickness and morphology in ASD

There is significant heterogeneity in the literature with respect to cortical thickness and morphology in ASD, with at times seemingly contradictory results depending on the age, IQ, and clinical severity of the study population.

In a very young group of patients with ASD, cortical volume, and surface area (but not thickness) were found to be increased compared to controls at the age of 2 years. The rate of cortical growth between ages 2 and 5 years did not differ between groups, further implicating the prenatal and early postnatal periods as central to disease pathogenesis (53).

In slightly older age groups, many authors have observed evidence of exaggerated cortical thinning in ASD. For example, Hardan et al. (54) demonstrated that children with ASD ages 8–13 years had increased cortical thickness, particularly in the temporal lobe, as compared to aged matched controls. The small sample size (*n* = 17 cases), however, precluded co-variation for IQ, or analysis of age-related interactions (54). Longitudinal imaging 2-years later on seemingly the same cohort, showed that those with a diagnosis of ASD underwent exaggerated cortical thinning compared to controls, and that the degree of thinning correlated with the severity of symptoms. Differences, however, were mostly non-significant after controlling for multiple comparisons and variation in IQ (55). In a comparable age group (6–15 years). Mak-Fan et al. (56) showed a similar pattern of increased cortical thickness, surface area, and gray matter volume in children with ASD at earlier ages (6–10 years), that then underwent exaggerated losses compared to controls, such that by 12–13 years of age, controls surpassed patients on all three measures (56). Wallace et al. (57), on the other hand, found baseline *deficits* in cortical thickness for adolescents with ASD, but also observed exaggerated rates of cortical thinning during adolescence and early adulthood (57). In the same study population, no differences in overall surface area were noted, but more overall gyrification in the ASD group, particularly in the occipital and parietal regions was observed. Both groups showed a decline in gyrification overtime (58).

On the other hand, several authors have noted deficits in cortical thinning in ASD. Looking over a wide age range, Raznahan et al. (59) used cross-sectional MRI data from 76 patients with ASD (primarily Asperger’s syndrome) and 51 controls from ages 10 to 60 years to study the effects of age on cortical thickness and surface area. While surface area was relatively stable and comparable between both groups, they found significant differences with respect to cortical thickness. Typically developing individuals had greater cortical thickness in adolescence, which thinned steadily overtime. Individuals with ASD had reduced cortical thickness early in life, which underwent relatively little cortical thinning overtime, such that by middle age, they had surpassed their typically developing peers (59). ASD associated deficits in expected age-related cortical thinning during adolescence and adulthood has been shown in several other studies as well, both diffusely and in specific subregions (60, 61).

Recently, Ecker et al. (62) sought to tease apart the relative contributions of cortical thickness and cortical surface area to overall differences in cortical volume in a group of adult males (mean age of 26 years) with ASD compared to controls. While total brain volume and mean cortical thickness measurements were not significantly different between the two groups, several regional clusters emerged with both increased and decreased cortical volumes. The authors found that these relative differences were accounted for by variability primarily in cortical surface area, and less so from cortical thickness. As well, differences in cortical thickness/surface area were largely non-overlapping, and were deemed to be spatially independent from each other (62).

As in COS, several groups have aimed to combine the predictive power of multiple measurements by applying mathematical algorithms to neuroimaging data. Ecker et al. (63), for example, included five parameters (cortical convexity, curvature, folding, thickness and surface area) in their support vector machine analytic approach. These combined measurements were able to correctly classify patients with ASD (*n* = 20) and controls (*n* = 20) with 80–90% specificity and sensitivity, with cortical thickness being the most predictive measurement. This approach also demonstrated proof of principle in separating patients with ASD from patients with ADHD, despite the small sample size, and lack of reproduction in a separate group of patients with ASD from which the algorithm was generated (63). Similarly, Jiao et al. (64) incorporated cortical thickness and volume data from children with ASD and controls (ages 7–13) into a machine-learning model with the aims of predicting presence or absence of ASD. One algorithm was able to predict diagnostic stratification with 87% accuracy based on cortical thickness measurements. The most predictive regions included both areas of decreased cortical thickness (in the left pars triangularis, orbital frontal gyrus, parahippocampalgyrus, and left frontal pole) and increased cortical thickness (left anterior cingulate and left precuneus) (64). Again, the case control design was not representative of true population prevalence, precluding calculation of positive predictive values.

##### Summary and comparison

In ASD, a small number of studies support a pattern of very early overgrowth in cortical surface area and volume (<2 years of age), which is immediately followed by cortical dysmaturation throughout childhood and adolescence, with evidence suggesting both exaggerated and impaired cortical thinning, depending on the study. Changes in cortical thickness and surface area seem to occur in non-overlapping regions. In COS on the other hand, cortical thickness is reduced diffusely in childhood, although data from very young patients (<8 years) are lacking. During adolescence, reductions in cortical thickness become more localized to frontal regions, although less has been written about the specific rates of cortical thinning in this patient group.

### Regions of interest

Studies seeking out and investigating specific regions of interest in both COS and ASD have employed several different approaches. On the one hand, a general approach simultaneously comparing dozens of regions of interest or thousands of specific points in the absence of an *a priori* defined hypothesis has been used to survey for areas associated with the greatest differences between patient and control samples, and can help guide future areas of research. On the other hand, a predefined hypothesis regarding volumetric differences in a particular region allows optimization of statistical power, to more precisely elucidate candidate regions.

#### Regions of interest in COS

A meta-analysis of studies conducted in adult onset schizophrenia patients describes global deficits in volume, most consistently in the left superior temporal gyrus and the left medial temporal lobe (65). Looking specifically at COS, in the NIMH cohort, an automated and longitudinal analysis of over 40,000 points across the cortical surface found that the superior and middle frontal gyris showed the greatest overall reduction in cortical thickness compared to controls (46). In a different sample COS population from UCLA, specific analysis of the right posterior superior temporal gyrus (Wernicke’s area, involved in verbal comprehension), found volume to be *increased* in this region (66). Investigations conducted by the same group on the anterior cingulate gyrus, a central and highly connected structure in the prefrontal cortex involved in many functions including error monitoring, yielded volume reductions (67).

Hypothesis driven approaches in the NIMH-COS cohort have been able to identify specific regional volume deficits as well. The insular cortex, for example, has been implicated in schizophrenia, given its role in distinguishing self from non-self, in visceral somatosensory interpretation, in processing of emotional experiences, and in salience. Patients with COS were found to have smaller insular volumes, whereas COS-siblings and controls were not statistically different, suggesting reduced insular size as an indicator of disease state. Additionally, level of functioning and severity of symptoms correlated with insular volume (68).

The cerebellum, classically understood to be involved in motor coordination and planning, has been implicated in schizophrenia given its association with learning and cognition. In longitudinal data from the NIMH cohort, smaller overall and regional cerebellar volumes were detected in affected individuals, with siblings falling between patients and controls on various measures (69).

Regarding subcortical structures, enlargement of the caudate (70) has been shown. In the limbic system, increased amygdala volume (71), but volume loss in the hippocampus and fornix (72, 73) has also been found in COS.

#### Regions of interest in ASD

Brain regions proposed to play a role in social cognition, communication, and “theory of mind” have been a focus of investigation in ASD. The region of the temporoparietal junction in particular, is thought to be central to the integration of social information and empathy, as well as selective attention to salient stimuli (74). Thinning of several areas in the temporoparietal region, particularly on the left side, has been shown in children, adolescents, and adults with ASD (38, 57, 59, 61, 75).

The orbital frontal cortex, in the ventromedial prefrontal region, is thought to play a role in sensory processing, goal directed behavior, adaptive learning, and attachment formation (76). Patients with autism, despite increased overall cortical thickness in the frontal region, have been shown to have specific deficits in cortical thickness (38), volume, and surface area (62) in the orbital frontal cortex, which correlated with symptoms severity (62). Other frontal lobe structures showing reduced cortical thickness in ASD include the inferior and middle frontal gyri, and the prefrontal cortex, depending on the study (38, 64, 77).

The anterior cingulate is a highly connected part of the social brain network situated along the medial aspect of the frontal cortex. Its role in self-perception, social processing, error monitoring, and reward based learning has been described (78). Relative increases (60, 64) and decreases (62, 75, 77) in volume and thickness of the anterior cingulate have been shown in ASD. Given that different regions may grow at different rates in individuals with ASD vs. controls (60, 61), variation in the age and distribution of study populations may account for some inconsistencies.

Volume deficits in the insular cortex have been demonstrated in young adults with pervasive developmental disorders (79). In adults with ASD, those who had a history of psychotic symptoms also demonstrated reduced insular volumes, particularly on the right side, as well as reduced cerebellar volumes (80).

Looking at subcortical structures, the caudate has been shown to be enlarged in ASD, across whole brain volumetric meta-analyses (81–83), and in targeted ROI analysis, even after controlling for confounding medication administration (84). Volume loss in the putamen has been shown across whole brain meta-analyses in adults with ASD (81, 83, 85), but enlargement of the putamen has also been observed in younger populations (86). In the amygdala, volume losses emerge across whole brain meta-analytic approaches (83, 85, 87), but volume gains are noted in younger patient groups as well (88). From a functional perspective, enlargement of the caudate may be associated with repetitive or self-injurious behavior (89–92), while volume loss in the amygdala may pertain to impaired emotional perception and regulation (93).

##### Summary and comparison

Volume losses have been noted in some overlapping prefrontal regions in both ASD and COS, particularly along the middle frontal gyrus. The anterior cingulate is also implicated in both conditions, although bidirectional changes in volume have been noted in ASD, depending on age of study participants. The area of the temporal-parietal junction shows volume loss in ASD, and was an area strongly predictive of diagnosis in group of individuals with COS (discussed in see Cortical Thickness and Morphology in COS). The insula is implicated in patients with COS, and in those with ASD who have comorbid psychotic symptoms. Looking at deep structures, both conditions are associated with volume gains in the caudate, which may pertain to repetitive behaviors, or concomitant neuroleptic treatment.

## Structural White Matter Analysis (Volumetric Analysis and Diffusion Tensor Imaging)

Magnetic resonance imaging analyses that incorporate diffusion measurements allow for further sub-characterization of white matter microstructure, above volumetric differences. The diffusion of water molecules is measurable with MRI technology, and the magnitude and direction of diffusion within each individual voxel can be modeled mathematically with vector algebra. Axial diffusivity (AD) is the measurement of diffusion occurring *parallel* to white matter fibers; increased AD occurs in diseases involving axonal degeneration, and is thought to reflect both the integrity and density of axon structures. Radial diffusivity (RD) on the other hand, is a measurement of diffusion occurring *perpendicular* to the white matter fibers; it is used as a measure of myelination, and is increased in demyelinating diseases. Mean diffusivity (MD) (also known as the apparent diffusion coefficient, ADC) is a measure of average diffusion in absence of a directional gradient (94).

A summary ellipsoid vector incorporating the overall spherical nature of the combined vectors is termed “fractional anisotropy” (FA). A perfectly “isotropic” solution (FA = 0), such as free water, contains molecules that diffuse freely in all directions, whereas an anisotropic solution (i.e., a white matter fiber bundle) would restrict diffusion in one direction resulting in an elongated ellipsoid and FA values closer to 1. In white matter tract analysis, increased FA is thought to be a sensitive but not specific measure of fiber myelination, the integrity of cell membranes as well as the diameter of the fibers (95). Typically developing individuals show age related increases in FA and decreases in MD throughout development, in keeping with increasing white matter maturation (96). As in gray matter analyses, DTI can be applied to the whole brain in a voxel-based approach, or alternatively, specific regions of interest can be investigated with this method. Along these lines, specific anatomic white matter tracts can be reconstructed and analyzed from DTI data, in a method known as tractography. DTI data can also be transposed onto a common FA template, in tract-based spatial statistics (TBSS) (97).

Magnetic resonance imaging data collected in the absence of diffusion measurements can still be utilized in studying white matter integrity and growth. Similar to gray matter analysis, simple volumetric studies on white matter structures have been employed. Alternatively, 3D mapping of volumetric changes in white matter tracts via tensor-based morphometry (TBM) has been validated as a method of studying white matter development over time. In brief, TBM applies initial and follow up scans to a standardized brain template to ensure precise anatomical alignment. Next, an elastic-deformation algorithm is used to calculate the specific degree of volume expansion in a set area, represented by an expansion factor called the “Jacobian determinant.” Growth rates are calculated by comparing the Jacobian determinant measures across patient and control samples.

### White matter analysis in COS

The corpus callosum is the largest white matter structure in the human brain, and is central for connectivity and relay of information between hemispheres. Deficits in the corpus callosum have been inconsistently demonstrated in adult onset schizophrenia populations (95). In a longitudinal analysis of children and young adults with COS, differences in the midsagittal area of the splenium of the corpus callosum emerged around age 22, with patients having significantly smaller structures (98). Later analysis looking at volumetric differences in subsections of the corpus callosum revealed no differences between NIMH-COS patients, their siblings and controls with respect to overall volume, and/or volume change over time (99).

Comparison of whole brain TBM data between 12 patients with COS and 12 age matched controls followed over a 5-year interval revealed aberrant white matter development between ages 13 and 19 years. Specifically, at baseline MRI, patients had a 15% deficit in white matter volume in the frontal regions. At follow up, control patients showed an average of 2.6% growth in white matter per year, while COS patient had only 0.4% white matter growth per year. The white matter deficits in the COS sample seemed to progress in a front to back pattern, opposite to previous findings regarding gray-matter deficits, but consistent with expected growth patterns in healthy adolescent brains (100). Unaffected siblings of children with COS showed delayed white matter growth at younger ages (<14 years) but not at older ages (14–18 years) as measured by TBM. Delayed white matter growth was most significant in the parietal regions for siblings, but normalized by age 18 (101).

There are relatively few DTI studies in specific COS populations. Clark et al. (102) found no significant differences in FA diffusely between 18 children and adolescents with COS, and 25 controls. Of note, five COS patients had a comorbid diagnosis of ASD, of which four were tested as having a linguistic impairment. Increased RD and AD was noted for patient vs. control groups in several white matter tracts (see Table 1). Increases in RD and AD in these regions were explained primarily by the presence of a linguistic impairment, and not the diagnosis COS, however (102).

**Table 1:** **Summary of white matter findings in ASD and COS**.

	COS vs. controls		ASD vs. controls	
	White matter volume in COS	DTI in COS	Meta-analysis on white matter volume in ASD	Meta-analysis on DTI in ASD
Study	(160); (99); (98)	(102)	(109)	(110)
Mean age of patient group	(160) 14.1–18.7; (99) 17.3; (98) 14.8	14.7	21.4	15.2
Whole brain white matter	↓(160)	ND FA	ND	–
Corpus callosum	↓(98); ND (99)	ND FA; ↑RD/AD in LI	↓	↓FA; ↑MD
Superior longitudinal fasciculus	–	ND FA; ↑RD/AD in LI (L)	–	↓FA (L); ↑MD
Arcuate fasciculus	–	ND FA	↑	–
Inferior longitudinal fasciulus	–	ND FA; ↑RD/AD in LI (L)	–	ND FA
Inferior fronto-occipital fasciculus	–	ND FA; ↑RD/AD in LI (L)	↑	ND FA
Cingulum	↓(160)	ND FA	↓	ND FA
Uncinate fasciulus	–	ND FA	↑	↓FA (L); ND MD

*Note that for ASD, significant findings are reported from meta-analyses only. COS, childhood onset schizophrenia; ASD, autism spectrum disorder; DTI, diffusion tensor imaging; ND, no difference; FA, fractional anisotropy; RD, radial diffusivity; MD, mean diffusivity; AD, axial diffusivity; L, left side; R, right side; LI, COS patients with language impairment*.

There is a growing body of literature, however, on diffusion tensor imaging in adult onset schizophrenia and early-onset schizophrenia (EOS: defined as symptom onset prior to age 18 years). Findings investigating these patient groups are summarized in several reviews (103, 104). Given the paucity of literature applying DTI in COS, some conclusions may be extrapolated from the early-onset schizophrenia literature; therefore they will be discussed briefly.

In general, while results have varied, the corpus callosum, superior and inferior longitudinal fasciculus, cingulum, and the uncinate fasciculus have been suggested as areas most affected with respect to white matter integrity as measured by decreases in FA (103, 104). Some studies have attempted to correlate DTI findings with symptomatology. Ashtari et al. (105), for example, found decreased FA in the left inferior longitudinal fasciculus was more pronounced for EOS patients with a history of visual hallucinations (105). As in volumetric imaging, studies that incorporate analyses for age effects provide evidence of dynamic white matter abnormalities as well, in EOS. For example, FA in the anterior cingulate region increased with age in the healthy control population, but decreased with age in the early onset psychosis population (106). Similarly, patients with EOS showed decreased FA in parietal regions, while patients with adult onset schizophrenia had findings localizing to the frontal, temporal, and cerebellar regions (107).

### White matter analysis in ASD

Earlier volumetric analyses suggested a pattern of accelerated of white matter volume and growth in younger children, particularly in the frontal regions, but that adolescents with ASD had similar or reduced white matter volume compared to controls (108). Meta-analysis of 13 VBM studies on white matter volume found no differences globally in white matter volume, and no differences between child/adolescent groups and adults groups, although no studies included very young children (<6 years). Some regional differences emerged, however (109) (see Table 1).

With respect to diffusion tensor imaging, a recent systematic review and meta-analysis, combining DTI data from 14 studies, including both children and adults with ASD, summarized some areas of consensus and heterogeneity in the literature. In summary, decreased FA was most consistently demonstrated in the corpus callosum, left uncinate fasciculus, and left superior longitudinal fasciculus of individuals with ASD. Mean diffusivity was increased in the corpus callosum, and bilaterally in the superior longitudinal fasciculus (110). This meta-analysis included data from ROI and tractography studies only, however, excluding whole brain TBSS and voxel-based analyses. A recent literature review on DTI in ASD by Travers et al. (97), identified decreased FA, increased MD, and RD as the most common finding across methods, with the corpus callosum, cingulum, arcuate fasciculus, superior longitudinal, and uncinate fasciculus showing the greatest differences (97).

Most imaging studies in autism to date, as well as those included in the above-described meta-analyses, have been conducted in older children, adolescents, or adults. In these age groups, decreased FA and increased MD have been repeatedly documented in many white matter regions. The specific rate of change in white matter markers, as well as the effect of age on white matter maturation seems to vary by study, however. For example, Mak-Fan et al. (56) showed RD and MD measurements stayed stable between the ages 6 and 14 years in subjects with ASD, while control subjects showed expected decreases with age (111). Ameis et al. (112) found the between group differences in RD, AD, and MD, but not FA, which were more pronounced in childhood than in adolescence (112).

Few studies have been conducted in very young children, however, and less consistency emerges in the data from this age range. Contrary to literature in older populations, Weinstein et al. (113), reported that FA was *greater* for children ages 1.5–6 years with ASD compared to controls in the areas of the corpus callosum, superior longitudinal fasciculus, and cingulum. Differences in FA were attributable to decreased RD, while AD was the same between cases and controls (113). Similarly, Ben Bashat et al. (114), found evidence of accelerated white matter maturation marked by increased FA and reduced displacement values in a small sample of children with ASD ages 1.8–3.3 years, most prominently in frontal regions (114). Abdel Razek and colleagues (115), found ADC scores to be greater for preschool children with ASD in several regions, which correlated with severity of autistic symptoms as measured by the childhood autism rating scale (115). Walker et al. (116) on the other hand, found that 39 children between ages 2 and 8 years with ASD had decreased MD and FA compared to controls, accompanied by an attenuated rate of increase in FA, as well an accelerated rate of decreased MD compared to controls (116). Longitudinal data looking at high risk infants found evidence of higher FA at 6 months in children who were subsequently diagnosed with ASD, but that they had then had a slower rate of change such that by 24 months typically developing children had surpassed them in this measure (117).

For most studies, although differences have been statistically significant for certain regions, the magnitude of these differences has been quite small, on the range of 1–2%, thus limiting the predictive ability of any individual measurement. Lange et al. (118) generated a discriminant function that was able to distinguish between individuals with and without ASD with 94% sensitivity, 90% specificity, and 92% accuracy, by combining the predictive ability of DTI data points centered primarily around the superior temporal gyrus and the temporal stem. The sensitivity and specificity was reproduced in a replicate sample as well, however the case-control design was not reflective of true population prevalence, again precluding inferences regarding predictive ability in a real life clinical setting (118).

Emerging efforts have tried to correlate neuroimaing findings to functional and behavioral outcomes. For example, increased MD in the superior longitudinal fasciculus correlated with degree of language impairment in children and adolescents (119). Increase FA and decreased RD in the arcuate fasciculus correlated with greater language abilities in another group of children with ASD (120). Similarly, lower FA in the dorsal lateral prefrontal region was associated with increased social impairment in a group of children with ASD in Japan (121). Attempts to identify structural deficits in areas involved socio-emotional processing have yielded mixed results as well. Further focus on understanding the functional connectivity between distant regions is described in the next section.

#### 

##### Summary and comparison

White matter development in COS patients compared to controls appears marked by global deficits in white matter volume and decreased rates of white matter growth/integrity in adolescence, although the specific chronology, most affected regions and the relation to symptoms continues to be explored. In ASD, meta-analyses suggest no differences overall in white matter volume in adults, although early white matter volumetric overgrowth may occur in younger patient samples. Looking at specific white matter regions, volume losses have been noted in both ASD and COS in the corpus callosum and cingulum. In both conditions, decreased white matter integrity as measured though DTI has been observed in the superior longitudinal fasciculus, which may pertain to comorbid language impairments.

## Functional Connectivity

While imaging of white matter tracts through techniques like DTI permits the quantification of structural connectivity between regions, *functional* connectivity requires *in vivo* analysis of brain activation. Functional magnetic resonance imaging (fMRI) measures regional changes in blood oxygen level dependent (BOLD) signaling, given the subtle differences in magnetic field strength between oxygenated and deoxygenated blood. Brain activation patterns may be analyzed in subjects at rest (termed resting state) or during a specific cognitive or behavioral task performed in an MRI scanner. Data can be analyzed with respect to a specific region of interest (seed technique), where connections to and from an *a priori* defined region are studied. Alternatively, independent component analysis (ICA), or similar techniques, look at overall activation patterns across all regions, and can comment on patterns in functional networks (i.e., default mode network, salience network). Data from functional neuroimaging studies are often analyzed using graph theory. In this approach, the relationship between certain areas of central activation (termed “nodes”) and the vectors of connectivity between nodes (termed “edges”) are described using discrete mathematics (122). Short-range connectivity (i.e., within a specific lobe, or to a neighboring lobe) and long-range connectivity between remote regions can be quantified in this manner.

### Functional connectivity in COS

Two separate analyses in the NIMH cohort of COS have suggested exaggerated long-range connectivity, and impaired short-range connectivity, in keeping with a hypothesis of exaggerated synaptic pruning. Resting state fMRI data was used to graph the connectivity between 100 regional nodes for 13 patients and 19 controls. Data showed that patients with COS had signals that were less clustered with more disrupted modularity marked by fewer edges between nodes of the same module. On the other hand, they showed greater global connectedness and greater global efficiency (123). Subsequent analyses with a slightly larger sample again found reduced connectivity at short distances and increased connectivity at long distances for patients with COS compared to controls on resting state fMRI. Relative to healthy controls, patients with COS had several regions in the frontal and parietal lobes that were “nodes” of over-connectedness with respect to long-range associations (124). White et al. (125) on the other hand, interpreted an opposite pattern from a study using a visual stimulus to analyze connectivity in the occipital lobe of children and adolescents with early onset schizophrenia (125). Similarly, structural connectivity analysis in neonates at high risk for schizophrenia found decreased global efficiency, increased local efficiency, and fewer nodes and edges overall compared to control infants (126).

### Functional connectivity in ASD

In ASD on the other hand, there is an abundance of recent literature on functional connectivity. An emerging hypothesis suggests that frontoparietal under connectivity in ASD results in reduced “bandwidth” in long-range circuits [reviewed by Just et al. (127)]. Some propose that this coincides with local increases in connectivity within a specific lobe, resulting in a failure to integrate and regulate multiple sources of information (128). This hypothesis is consistent with structural white matter deficits in long-range association fibers, as well as structural patterns in gray matter showing increased local, but deficits in global modularity (129).

With respect to functional analyses, impaired synchronization, and under connectivity between large-scale networks has been shown in fMRI studies incorporating various task-based assessments, including those pertaining to language comprehension and auditory stimuli (130–132), executive functioning (133), visual spatial processing (134), and response to emotional cues (135, 136). Under connectivity has not been the only finding however, with many functional MRI studies showing evidence of increased connectivity or altered developmental trajectories with respect to integrated neural networks (137–139). For example, a recent meta-analysis of fMRI studies found greater activation in children with ASD in response to a social task in certain specific regions (i.e., in the left-precentral gyrus) but relative under activation compared to controls in other areas (superior temporal gyrus, parahippocampal gyrus, amygdala, and fusiform gyrus). In adults with ASD, activation was greater in the superior temporal gyrus, but less in the anterior cingulate during social processing (140).

The literature is also divided with respect to functional neuroimaging in resting state MRI, in the absence of any particular stimulus or task. Some have proposed that methodological issues may be contributing to observed inconsistencies (141). While hypoconnectivity seems most prevalent in the literature, [Ref. (142, 143); reviewed by Uddin et al. (144)], Uddin et al. (144) observed long-range hyperconnectivity via ICA across remote regions in 20 children ages 7–12 years with autism compared to controls. Hyperconnectivity was noted to involve the default mode network, frontotemporal, motor, visual, and salience networks. Hyperconnectivity of the salience network (which involves the anterior cingulate and insula) was most predictive of the diagnosis of ASD and was able to discriminate between cases and controls with 83% accuracy, a finding that was reproduced in a separate image dataset (145). Other resting state fMRI studies have also observed mixed patterns, which vary by region, network, and by age of the sample (146, 147).

The literature in very young patients with ASD is relatively sparse but seems to suggest altered developmental trajectories for affected children beginning at very young ages. A recent publication observed increased functional connectivity at 3 months, which disappeared by 12 months in high risk infants (148). Alternatively, Redcay and Courchesne (139) found increased connectivity between hemispheres in 2–3 year old children with ASD compared to chronological age matched controls, however the opposite pattern emerged when they were compared to mental age matched controls (139). Dinstein et al. (132) observed hypoconnectivity between hemispheres and in language regions in toddlers with ASD in response to auditory stimuli (132).

A recent review article by Uddin et al. (144) summarizes the literature to date with respect to resting state functional connectivity analyses. While intrinsic connectivity and seed-based analyses across 17 published studies suggest both hyper- and hypo-connectivity, Uddin and colleagues propose that the developmental age of the sample may be one explanatory factor with respect to variability in results. They describe a hypothesis in which increased functional connectivity in prepubescent children with ASD as compared to their peers is then met with altered maturational trajectories such that adults with ASD seem to have reduced connectivity compared to controls (144).

A recent publication put forth by a data sharing initiative entitled “autism brain imaging data exchange” (ABIDE) proposes to remedy disagreement in the literature through a large-scale international collaboration combining 1112 resting state fMRI scans. Analysis of 360 male subjects with ASD compared to controls found hypo connectivity in cortical networks but hyper connectivity in subcortical networks. They also identified localized differences in connectivity in certain regions, including the insula, cingulate, and thalamus. They did not perform specific analyses looking for age-associated differences, however, given that the majority of included participants were adolescents or adults (146).

#### 

##### Summary and comparison

There are only a handful of studies looking at functional connectivity in COS, but data from fMRI suggest a pattern of increased long-range connectivity, with disrupted short-range connectivity, in keeping with pathology of exaggerated synaptic pruning. In comparison, data from fMRI in ASD suggest to some extent an opposite pattern, with increased local but decreased global connectivity. fMRI data sharing between research centers reveal hyperconnectivity in subcortical networks, and hypoconnectivity in cortical networks in adult males with ASD. Smaller studies in younger age groups suggest important age effects regarding the connectivity hypothesis as well, with younger children with ASD seemingly showing more “over-connectedness” than adults.

## Discussion

This review compares and contrasts neuroimaging findings in ASD and COS. Overall, across volumetric, structural, and functional neuroimaging data, there arises evidence for a dynamic changes in both conditions. In ASD, a pattern of early brain overgrowth is seemingly met with dysmaturation in adolescence, although the literature in this regard is far from certain. Functional analyses have suggested impaired long-range connectivity as well as increased local and/or subcortical connectivity, which may also progress with age. In COS, global deficits in cerebral volume, cortical thickness, and white matter maturation seem most pronounced in childhood and adolescence, and may level off in early adulthood. Deficits in local connectivity, with increased long-range connectivity have been proposed, in keeping with exaggerated cortical pruning; however the opposite has also been shown. Symptom and neuroimaging overlap across conditions was illustrated via a meta-analysis of fMRI data in both schizophrenia and ASD, which identified shared deficits in regions involved in social cognition (149).

The significance of these findings is tempered, however, by heterogeneity in results across other pediatric onset neurodevelopmental disorders. In ADHD for example, longitudinal MRI analyses in children suggest overall reduced cortical thickness prior to the onset of puberty (158) with peak cortical thickness and onset of cortical thinning occurring at later ages (159). In the future, clinical neuroimaging must be able to identify not only the presence of aberrant neurodevelopment, but also be able to discern across overlapping conditions.

While there is heterogeneity in the literature in both conditions, findings regarding COS at times appear more consistent. It is important to note that, given the rarity of this condition, these findings emerge from relatively few research samples, and are derived primarily from data collected from the same population of individuals. In ASD on the other hand, there has been an international explosion of investigation at numerous institutions, across ages, IQ ranges, and diagnostic severity, which has resulted in at times seemingly contradictory results. A call for collaboration (150) has been met with a first international compilation of neuroimaging datasets, which has helped to clarify some discrepancies in the literature with respect to fMRI (146). Going forward, ongoing collaboration to facilitate large scale, prospective, longitudinal neuroimaging studies, will be necessary to separate signals from noise in these complex and heterogeneous diseases. A focus on genetic subtypes may help to unite synapse pathology with neuroimaging findings and network dysfunction, to permit some degree of hypothesis generation with respect to molecular pathogenesis.

In ASD, for example, a loss of inhibitory control leading to exaggerated growth, premature cortical thinning, and then early stabilization of cortical structures has led some to suggest that overall the developmental curve has been “shifted to the left” along the time axis in this condition, with respect to brain maturation (75, 151). Current genetic investigations suggest alterations in structural scaffolding at the excitatory synapse could be contributory in ASD (152). Single gene disorders associated with autism may shed light on underlying final common pathways (153). Fragile X syndrome (FXS), for example, is a genetic condition comorbid with ASD in 20–30% of cases (154). Individuals afflicted with this condition have dysfunction or absence of the fragile X mental retardation protein (FMRP). FMRP is now understood to play a critical role in regulation of protein synthesis at the excitatory synapse, and without it, exaggerated receptor cycling and dysfunctional neuroplasticity can results (153). A similar mechanism in idiopathic ASD would hypothetically results in a loss of inhibitory control on expected maturational changes, uncoupling the structural and temporal timeline of synaptic neurodevelopment.

In schizophrenia, exaggerated synaptic pruning has been a long held hypothesis with respect to an etiology (25), which is consistent with aspects of the neuroimaging literature in COS. On the other hand, a small study in high risk infants suggests enlarged cerebral volumes may exist early in life, implying that some type of early dysregulated growth may be at play in this condition as well, similar to the process occurring in ASD (27). Investigations in 22q11.2 deletion syndrome (DS), a genetic disorder associated with schizophrenia in 20–25% of cases (155), permits longitudinal and prospective analysis of children at high risk for schizophrenia. Interestingly, MRI data collected in children as young as 6 years old with 22q11.2 DS found early increases in cortical thickness and deficits in cortical thinning in preadolescence, which are then met with exaggerated cortical thinning during adolescent years. Patients who subsequently developed schizophrenia indeed had more exaggerated deficits in cortical thickness (156).

In studies recruiting adolescents, it is difficult to tease out the possible influence of confounders such as substance abuse on both clinical and radiologic findings. While comorbid substance abuse is common in adult onset schizophrenia populations (occurring in 50–80% cases), the rate of substance abuse in COS, while presumed lower, has not been described (157). Ongoing study of clinical, environmental, and cultural confounding factors in both ASD and COS is needed.

Many investigators have sought to use neuroimaging protocols as predictors of diagnosis in case-control studies. The accuracy, sensitivity, and specificity of these analyses have on average ranged between 60 and 90%, and some groups have been able to reproduce high levels of diagnostic accuracy in separate patient samples. The clinical utility of these algorithms, however, remains uncertain in the absence of their application to populations reflecting realistic disease prevalence (i.e., positive predictive values are low or not reported). The development of clinically useful, cost-effective wide scale diagnostic tests for neurodevelopment conditions remains a common goal, and several groups have initiated prospective trials on high risk patient populations which may perhaps yield some hopeful results in the next decade.

## Authors Contribution

Danielle A. Baribeau authored the manuscript. Evdokia Anagnostou developed the research topic, provided guidance, editing, and supervision.

## Conflict of Interest Statement

Danielle A. Baribeau has no financial conflicts to disclose. Evdokia Anagnostou has consulted to Seaside Therapeutics and Novartis. She has received grant funding from Sanofi Canada. The Guest Associate Editor – Stephanie Ameis – declares that, in spite of her adjunct affiliation with the same institution as authors Danielle A. Baribeau and Evdokia Anagnostou, the review process was handled objectively and no conflict of interest exists.
